# Artificial Intelligence for Medical Diagnostics—Existing and Future AI Technology!

**DOI:** 10.3390/diagnostics13040688

**Published:** 2023-02-12

**Authors:** Mugahed A. Al-Antari

**Affiliations:** Daeyang AI Center, Department of Artificial Intelligence, College of Software & Convergence Technology, Sejong University, Seoul 05006, Republic of Korea; en.mualshz@sejong.ac.kr

We would like to express our gratitude to all authors who contributed to the Special Issue of “*Artificial Intelligence Advances for Medical Computer-Aided Diagnosis*” by providing their excellent and recent research findings for AI-based medical diagnosis. Furthermore, special thanks are extended to all reviewers who helped us to process an article in this Special Issue. Finally, we would like to express our deep and warm gratitude and respect to the editorial members working day and night on this Special Issue, providing the recent AI-based research studies to enrich the AI medical knowledge for the fourth industrial revolution.

Medical diagnostics is the process of evaluating medical conditions or diseases by analyzing symptoms, medical history, and test results. The goal of medical diagnostics is to determine the cause of a medical problem and make an accurate diagnosis to provide effective treatment. This can involve various diagnostic tests, such as imaging tests (e.g., X-rays, MRI, CT scans), blood tests, and biopsy procedures. The results of these tests help healthcare providers determine the best course of treatment for their patients. In addition to helping diagnose medical conditions, medical diagnostics can also be used to monitor the progress of a condition, assess the effectiveness of treatment, and detect potential health problems before they become serious. With the recent AI revolution, medical diagnostics could be improved to revolutionize the field of medical diagnostics by improving the prediction accuracy, speed, and efficiency of the diagnostic process. AI algorithms can analyze medical images (e.g., X-rays, MRIs, ultrasounds, CT scans, and DXAs) and assist healthcare providers in identifying and diagnosing diseases more accurately and quickly. AI can analyze large amounts of patient data, including medical 2D/3D imaging, bio-signals (e.g., ECG, EEG, EMG, and EHR), vital signs (e.g., body temperature, pulse rate, respiration rate, and blood pressure), demographic information, medical history, and laboratory test results. This could support decision making and provide accurate prediction results. This can help healthcare providers make more informed decisions about patient care. The diversity of the patient’s data in terms of multimodal data is an optimal smart solution that could provide better diagnostic decisions based on multiple findings in images, signals, text representation, etc. By integrating multiple data sources, healthcare providers can gain a more comprehensive understanding of a patient’s health and the underlying causes of their symptoms. The combination of multiple data sources can provide a more complete picture of a patient’s health, reducing the chance of misdiagnosis and improving the accuracy of diagnosis. Multimodal data can help healthcare providers monitor the progression of a condition over time, allowing for more effective treatment and management of chronic diseases. Meanwhile, using multimodal medical data, Explainable XAI-based healthcare providers can detect potential health problems earlier, before they become serious and potentially life-threatening [1]. Moreover, AI-powered Clinical Decision Support Systems (CDSSs) could provide real-time assistance and support to make more informed decisions about patient care. XAI tools can automate routine tasks, freeing healthcare providers to focus on more complex patient care.

The future of AI-based medical diagnostics is likely to be characterized by continued growth and development as OpenAI [2]. More advanced AI technologies are being introduced into the research domain, such as quantum AI (QAI), to speed up the conventional training process and provide rapid diagnostics models [3]. Quantum computers have significantly more processing power than classical computers, and this could allow quantum AI algorithms to analyze vast amounts of medical data in real-time, leading to more accurate and efficient diagnoses. Quantum optimization algorithms can optimize decision-making processes in medical diagnostics, such as choosing the best course of treatment for a patient based on their medical history and other factors. Another concept is GAI or general AI, which is being used by different projects and companies, such as OpenAI’s DeepQA, IBM’s Watson, and Google’s DeepMind. The goal of GAI for medical diagnostics is to improve the accuracy, speed, and efficiency of medical diagnoses, as well as provide healthcare providers with valuable insights and support in the diagnosis and treatment of patients. By using AI algorithms to analyze vast amounts of medical data and identify patterns and relationships, general AI for medical diagnostics can transform the field of medicine, leading to improved patient outcomes and a more efficient and effective healthcare system. However, the development and deployment of AI in medical diagnostics are still in the early stages, and there are several technical, regulatory, and ethical challenges that must be overcome for the technology to reach its full potential. The first challenge is due to medical data quality and availability, where AI algorithms require large amounts of high-quality labeled data to be effective, and this can be a challenge in the medical field, where data are often fragmented, incomplete, unlabeled, or unavailable. Meanwhile, AI algorithms can be biased if they are trained on data that is not representative of the population they are intended to serve, leading to incorrect or unfair diagnoses. Another issue is about the use of GAI in medical diagnostics of a private and sensitive dataset, which raises some ethical questions, including data privacy, algorithmic transparency, and accountability for decisions made by AI algorithms. Even though some solutions with federated learning have recently been presented to solve such issues, the tool still needs more investigation to approve its capability for the medical research area. In addition, AI-based medical diagnostic tools are often developed by different companies and organizations, and there is a need for interoperability standards and protocols to ensure that these tools can work together effectively. AI-based techniques can analyze a patient’s medical history, genetics, and other factors to create personalized treatment plans, and this trend will likely continue to be developed in the future. However, AI-based medical diagnostics is an open research domain, and we highly recommend that researchers continue research to improve the final prediction accuracy and expedite the learning process. This will support the medical staff in hospitals and healthcare centers and even assist the industrial sector by providing novel smart solutions against epidemics or pandemics that suddenly appear and devastate communities worldwide.

## Data Availability

Not applicable.

## Abbreviations

GAI	General Artificial Intelligence.
XAI	Explainable Artificial Intelligence.
QAI	Quantum Artificial Intelligence.
ECG	Electrocardiogram.
EEG	Electroencephalogram.
EMG	Electromyography.
EHR	Electronic healthcare records.
MRI	Magnetic resonance imaging.
CT	Computed tomography.
DXA	Dual-energy X-ray absorptiometry.
